# Physical and technical performance in and after the worst-case scenario in matches of the Chinese Super League of soccer

**DOI:** 10.5114/biolsport.2025.142642

**Published:** 2024-09-06

**Authors:** Zuoming Fang, Zhaoyang Wang, Xueliang Li, Miguel-Angel Gómez, Hongyou Liu

**Affiliations:** 1 Department of Physical Education, Guangdong University of Education, Guangzhou, China; 2 School of Physical Education & Sports Science, South China Normal University, Guangzhou, China; 3 Department of Physical Education, Central South University, Changsha, China; 4 Faculty of Physical Activity and Sport Sciences, Technical University of Madrid, Madrid, Spain

**Keywords:** Peak demands, Most demanding scenario, CSL, High intensity running, Effective playing time

## Abstract

This study aims to investigate the changes in physical and technical performance of professional soccer players during effective playing time after the worst-case scenario (WCS) identified by the high-intensity running (HIR) distance using rolling average. A total of 576 matches (n = 13,298 observations) from the 2019 to 2021 season of the Chinese Super League (CSL) were analyzed by a video tracking system. Generalized mixed linear models were established to determine the mean changes in the value of 7 physical and 24 technical performance-related parameters in the effective playing time from the 5 min of WCS (Peak5) to the initial 5 minutes post-WCS (Post5). Results showed that: (1) For all the players in the Post5 total distance, HIR distance, and Sprint decreased by 16.6% (ES; ± 99%CL: 0.57; ± 0.04), 77.2% (2.78; ± 0.06), and 86.1% (2.11; ± 0.08), respectively. The number of efforts, average duration, average speed, and average length of HIR declined by 70.8% (2.26; ± 0.06), 31.7% (0.78; ± 0.05), 3.1% (0.52; ± 0.05), and 22.9% (0.83; ± 0.05), respectively; (2) In the Post5, a substantial decrement in the number (0.23; ± 0.03), average speed (0.32; ± 0.06) and average length (0.37; ± 0.06) of running with the ball, and average speed receiving the ball (0.5; ± 0.05) was observed for all players. While only trivial changes were detected in all the other technical performance-related parameters. It can be concluded that, in the Post5, there is a temporary decline in physical output and the physical-related technical parameters for players, however, there are no meaningful changes in other technical performancerelated parameters.

## INTRODUCTION

Soccer, as an intermittent team sport, involves various physical, technical, and tactical elements that influence player performance [1]. Players constantly shift between short bursts of high-intensity effort and extended periods of low-intensity activity in match [2, 3]. In this intermittent context, coaches face the challenge of designing specific training drills to prepare for the most intense phases of a game, often referred to as the ‘worst case scenario (WCS)’ [4, 5], or sometimes ‘peak demands’ [6], ‘most demanding passage’ [7], ‘peak locomotor demand’ [8], or ‘maximal intensity period’ [9]. Quantified player performance of WCS using different time windows in competition can serve as a benchmark for training, especially when combined with specific technical and tactical training targets, such as SSGs [9, 10].

Although the consensus [11–14] indicates a decline in physical output following a WCS across various time windows (1 min, 2 min, 5 min, 10 min), earlier studies have faced certain limitations. Some studies utilized fixed epochs to identify WCS, leading to under- or over-estimations of physical demands during WCS and subsequent periods [11, 12]. The current trend involves a rolling average method to accurately identify potential intense periods [14, 15]. Moreover, studies have shown that game interruptions can significantly amplify declines in match running performance, suggesting potential over-estimation of fatigue-induced performance decline [16]. However, the available research is scarce when analyzing the temporary decline in running performance excluding game interruptions. In addition, the analyzed parameters primarily pertain to distance and effort in running performance, such as distance covered in various speed zones and the number of accelerations and decelerations. However, there is less focus on other aspects of running performance, including the length, duration, and average speed of high-intensity running [5].

Furthermore, as a highly complexed system that integrates technical, tactical, physical, and psychological elements, soccer matches involve the interaction and mutual supplementation of all these factors [17, 18]. A temporary decline in physical output will inevitably lead to fluctuations in players’ technical and tactical performances. Therefore, an in-depth analysis of players’ WCS periods and their subsequent technical and tactical performances helps to understand the changes in different elements of the match. This information allows coaches to recreate peak match intensity during specific technical and tactical training sessions, optimizing players’ performance and reducing potential injury risks [19]. Nevertheless, the study of Carling and Dupont [20] was the unique which has reported the fluctuation of technical performance of professional soccer players after the WCS. They reported that the technical performance of professional soccer players showed no significant changes in the 5 minutes following peak-5 minutes of high-intensity running they had experienced. However, this finding is derived from observations of a restricted number of players from a single team, and the 5-minute epochs were evaluated using a fixed approach.

Another issue is that most of the published research on WCS has focused on European professional soccer players, with little attention given to Asian teams that are calling for more scientific feedback [13–15, 21]. In recent years, the Chinese Super League (CSL) has attracted many high-level coaches and players, significantly raising the league’s standard. Meanwhile, the introduction of advanced player tracking technologies has generated detailed match reports and accumulated a vast dataset of player performance metrics [22]. Hence, the CSL can serve as an ideal model for analysis.

Therefore, employing an amplified sample of players from CSL, this study aims: (i) to identify the WCS using a rolling average approach, and (ii) to examine the changes in physical and technical match performance in the effective playing time from the 5 min of WCS (Peak5) to the initial 5 minutes post-WCS (Post5) in professional soccer players. It is hypothesized that in the Post5, there will be a temporary decline not only in the total amount of players’ running output (distance and efforts), but also in the length, duration and speed of each high-intensity running (HIR) effort. Concurrently, related technical performance is expected to decrease. Additionally, it is hypothesized that changes in running performance and technical performance will vary depending on the player’s position.

## MATERIALS AND METHODS

### Participants and Data Collection

An observational approach was employed to assess the physical and technical performance of players in 576 matches spanning the 2019 to 2021 seasons of the CSL. The analysis focused exclusively on match data of outfield players who initiated and completed the entire match. This deliberate inclusion criterion aimed to prevent potential bias introduced by substitutes appearing towards the end of matches [23]. The final dataset comprised 13,298 observations from 970 players. These players were categorized into 5 tactical positions: central defender (CD, n = 214 players, 3,764 observations), full back (FB, n = 192 players, 2,580 observations), central midfielder (CM, n = 242 players, 3,414 observations), wide midfielder (WM, n = 135 players, 1,170 observations), and forward (FW, n = 187 players, 2,370 observations).

The performance data of the players for each match were captured using a multiple-camera computerized tracking system (STATS Pro^®^, Sport-Universal Process, Nice, France). The movement of players was observed during the match at a sampling rate of 25 Hz by cameras installed at the roof level [13]. The signals and angles generated by the sensors were sequentially turned into digital data and stored on computers for post-match analysis. All data were downloaded after the match using the appropriate proprietary software (STATS Viewer, version 3.6.0.3). The validity and reliability of the data collected by this tracking system have been previously verified [24–26]. Ethics committee approval for this study was obtained from the local university.

### Identification of worst-case scenarios

High-intensity running (HIR) efforts were first identified by the running speed threshold of above 19.8 km · h^−1^ [15, 27]. A rolling average approach was then applied to locate the 5 min with the most distance covered at HIR for each player, to identify the Peak5 and Post5. If Peak5 occurred at a time that is less than 5 min before the end of half-time or full-time of the match (extra time included), the data point would be removed from the analysis.

### Variables

Based on previous study of WCS analysis [15, 27], total distance (TD), distance covered (HIRD), efforts made (HIRE), average duration (AvdrtnHIR), average speed (AvspdHIR) and average length (Avlnth HIR) of HIR, and sprinting distance (Sprint, effort with speed greater than 25.2 km · h^−1^) were chosen to quantify the physical match performance of players in this study. Meanwhile, 24 variables (16 attacking-related variables, 2 transition-related variables, and 6 defendingrelated variables) were selected to represent the technical match performance of players (Table 1).

**TABLE 1:** Selected technical performance-related match-play variables.

Group	Events and variables: operational definitions
Variables related to attacking	Pass: a distribution action performed by a player to keep possession of the ball.

	Pass accuracy (%): successful passes as a proportion of total passes.

	Short pass: The player attempts to pass the ball to a teammate over a distance of less than 15 meters.

	Short pass accuracy (%): successful short passes as a proportion of total short passes.

	Long pass: The player attempts to pass the ball to a teammate over a distance of less than 25 meters.

	Long pass accuracy (%): successful long passes as a proportion of total long passes.

	Forward pass: an intentionally played ball from one player to another who is in the opponent’s half of the pitch.

	Forward pass accuracy (%): successful forward passes as a proportion of total forward passes.

	Running with the ball: deliberate movement of a player possessing the ball (no less than 3 touches).

	Average speed of running with the ball: average velocity of each running with the ball by a player during match play.

	Average length per running with the ball: average distance covered of each running with the ball by a player during match play.

	Individual possessions: number of individual players in controlled possession.

	Average speed at receiving the ball: the average running speed at which players move to receive a pass from a teammate each time.

	Average touches per individual possession: average number of touches per individual possession.

	Average time per individual possession: average duration of each possession.

	Shot: an attempt to score a goal, made with any (legal) part of the body, either on or off-target

Variables related	Ball regains: player’s actions (interception, picking up, won challenge) ending opponent’s ball possession and giving to transition a chance of making quick counterattack.

	Ball lost: player’s actions (inaccurate pass, lost challenge, etc.) that lead to losing ball possession.

Variables related	Duel: the number of attempts by a player from the opposing team to win possession of the ball on the ground or to defending aerial.

	Duel Successful (%): the number of successful duels as a proportion of total duels.

	Clearance: a player attempts to clear the ball up field or out of play, usually to relieve the pressure or danger faced by themselves or their team.

	Clearance Successful (%): the number of successful clearances as a proportion of total clearances.

	Tackle made: an attempt by a player to dispossess their opponent.

	Tackle successful (%): the number of successful tackles as a proportion of total tackles.

Variables in percent units (e.g., pass accuracy, duel successful, etc.) and already averaged (e.g., the average speed of HIR, the average speed of running with the ball, etc.) were analyzed at their raw values. The other variables were standardized to per-minute values, i.e., TD (m · min^−1^) = distance covered (m) in effective playing time / effective playing time (s) · 60.

### Statistical Analysis

The generalized mixed linear modelling was conducted in the academic version of the statistical analysis system (SAS^®^ OnDemand for Academics). Separate Poisson regressions were run in the modelling, with the values of each of the 7 variables related to physical performance and the 24 variables related to technical performance as dependent variables. Match location, match result, team strength, and quality of opponent were added as fixed effects (independent variables), while WCS was included as the main effect. Player names and team identities were included as random effects to account for repeated measures of players and teams. Match location, match result, and WCS were included as nominal predictor variables in the model. Match location (home, away, and neutral) and match result (win, draw, loss) were with three levels, while WCS was with two levels (Peak5, and Post5). Team strength and opponent strength were estimated by including the difference in the log of the end-ofseason ranks as a predictor [28].

The established models could properly estimate the mean values for each physical and technical performance-related parameter in the Peak5 and Post5 periods, controlling the effects of situational factors. The non-clinical magnitude-based inference was used to determine the changes in the mean of each parameter from the Peak5 to the Post5 period. Estimated magnitudes and their confidence limits were expressed in standardized units (standardization was achieved by this formula: (mean post5 – mean peak5)/SD Peak5), and were evaluated qualitatively using the following scale: < 0.2 trivial, 0.2~0.6 small, 0.6–1.2 moderate, 1.2–2.0 large, > 2.0 very large [29]. Effects were considered clear if the 99% confidence interval did not include both positive (> 0.2) and negative (< -0.2) values at the same time. Clear effects were reported with a qualitative probability that the true effect was substantial or trivial using the following scale: < 0.5% most unlikely, 0.5–5% very unlikely, 5–25% unlikely, 25–75% possibly, 75–95% likely, 95–99.5% very likely, and > 99.5% most likely [29].

## RESULTS

### Physical Performance

Table 2 presents the change in physical performance from Peak5 to Post5. As can be seen, when pooling all the players together, all the variables related to physical performance showed clear substantial decrement in the Post5 period. HIRD, HIRE, and Sprint showed a very large decrease, while TD, AvdrtnHIR, AvspdHIR, and AvlnthHIR showed a small decrement. A similar trend has been observed when dividing the players into different positions.

**TABLE 2:** Physical performance during effective playing time of peak5 and post5 for players (in)dependent of position.

Variables	ALL				CD			

	Peak5(m ± sd)	Post5(m ± sd)	%Change; ± 99%CI	ES; ± 99%CI	Peak5(m ± sd)	Post5(m ± sd)	%Change; ± 99%CI	ES; ± 99%CI
TD(m · min^−1^)	156 ± 51	130 ± 46	-16.6; ± 1.16	0.57; ± 0.04	145 ± 22	121 ± 20	-16.6; ± 1.01	1.18; ± 0.08
HIRD(m · min^−1^)	34 ± 19	7.6 ± 9.2	-77.2; ± 0.79	2.78; ± 0.06	28 ± 14	5 ± 7	-83.0; ± 1.22	3.62; ± 0.15
HIRE(m · min^−1^)	1.9 ± 1.1	0.54 ± 0.59	-70.8; ± 0.95	2.26; ± 0.06	1.5 ± 0.8	0.36 ± 0.44	-77.0; ± 1.56	2.84; ± 0.13
AvdrtnHIR (s)	6.3 ± 3.2	4.3 ± 2.5	-31.7; ± 1.51	0.78; ± 0.05	5.8 ± 3.2	3.8 ± 2.4	-34.3; ± 3.10	0.79; ± 0.09
AvspdHIR(km · h^−1^)	22.8 ± 1.4	22.1 ± 1.4	-3.1; ± 0.28	0.52; ± 0.05	22.8 ± 1.4	22.0 ± 1.4	-3.5; ± 0.59	0.57; ± 0.10
AvlnthHIR(m)	19.2 ± 6.1	14.8 ± 5.4	-22.9; ± 1.29	0.83; ± 0.05	19.2 ± 6.5	14.3 ± 5.6	-25.9; ± 2.70	0.90; ± 0.11
Sprint(m · min^−1^)	10.1 ± 10.9	1.4 ± 7.5	-86.1; ± 1.09	2.11; ± 0.08	8.8 ± 9.7	0.82 ± 8.3	-90.6; ± 1.74	2.50; ± 0.20

**Variables**	**FB**				**CM**			

	**Peak5(m ± sd)**	**Post5(m ± sd)**	**%Change; ± 99%CI**	**ES; ± 99%CI**	**Peak5(m ± sd)**	**Post5(m ± sd)**	**%Change; ± 99%CI**	**ES; ± 99%CI**

TD(m · min^−1^)	158 ± 69	126 ± 61	-20.0; ± 3.31	0.53; ± 0.10	164 ± 41	138 ± 38	-15.8; ± 1.79	0.69; ± 0.09
HIRD(m · min^−1^)	35 ± 21	8 ± 10	-77.2; ± 1.83	2.64; ± 0.14	34 ± 18	8 ± 9	-76.1; ± 1.53	2.80; ± 0.12
HIRE(m · min^−1^)	1.96 ± 1.1	0.57 ± 0.60	-70.8; ± 2.09	2.34; ± 0.14	1.9 ± 1.1	0.57 ± 0.60	-70.0; ± 1.89	2.21; ± 0.12
AvdrtnHIR (s)	6.3 ± 3.2	4.4 ± 2.6	-29.9; ± 3.46	0.72; ± 0.10	6.4 ± 3.1	4.5 ± 2.5	-28.9; ± 3.04	0.73; ± 0.09
AvspdHIR(km · h^−1^)	22.8 ± 1.4	22.2 ± 1.4	-3.0; ± 0.65	0.50; ± 0.11	22.6 ± 1.3	21.9 ± 1.3	-2.9; ± 0.52	0.51; ± 0.09
AvlnthHIR(m)	19.3 ± 6.1	14.9 ± 5.4	-22.4; ± 2.86	0.81; ± 0.12	19.1 ± 5.9	14.7 ± 5.2	-23.1; ± 2.47	0.86; ± 0.10
Sprint(m · min^−1^)	11.1 ± 11.0	1.6 ± 7.3	-85.6; ± 2.40	2.21; ± 0.19	8.8 ± 11.1	1.3 ± 8.7	-84.8; ± 2.53	1.79; ± 0.16

**Variables**	**WM**				**FW**			

	**Peak5(m ± sd)**	**Post5(m ± sd)**	**%Change; ± 99%CI**	**ES; ± 99%CI**	**Peak5(m ± sd)**	**Post5(m ± sd)**	**%Change; ± 99%CI**	**ES; ± 99%CI**

TD(m · min^−1^)	166 ± 27	138 ± 24	-16.5; ± 1.90	1.13; ± 0.14	152 ± 76	131 ± 71	-14.0; ± 4.35	0.31; ± 0.10
HIRD(m · min^−1^)	36 ± 16	10 ± 8	-72.9; ± 2.43	3.10; ± 0.21	37 ± 22	10 ± 13	-73.9; ± 2.35	2.38; ± 0.16
HIRE(m · min^−1^)	2.0 ± 0.9	0.67 ± 0.54	-65.9; ± 2.96	2.44; ± 0.20	2.0 ± 1.3	0.7 ± 0.8	-67.1; ± 2.90	1.84; ± 0.15
AvdrtnHIR (s)	6.6 ± 3.0	4.0 ± 2.1	-39.7; ± 4.13	1.17; ± 0.16	6.6 ± 3.0	4.5 ± 2.4	-31.0; ± 3.37	0.85; ± 0.11
AvspdHIR(km · h^−1^)	22.9 ± 1.3	22.2 ± 1.3	-2.7; ± 0.86	0.48; ± 0.16	23.0 ± 1.4	22.3 ± 1.4	-3.3; ± 0.65	0.55; ± 0.11
AvlnthHIR(m)	19.0 ± 5.5	15.0 ± 4.9	-21.0; ± 3.80	0.83; ± 0.17	19.1 ± 5.9	15.2 ± 5.3	-20.6; ± 2.97	0.75; ± 0.12
Sprint(m · min^−1^)	11.1 ± 11.0	1.9 ± 6.8	-82.7; ± 3.85	2.01; ± 0.25	12.5 ± 11.4	1.9 ± 7.0	-84.7; ± 2.42	2.29; ± 0.19

Note: all the values are estimated from the generalized mixed linear modelling. %Change = change in the mean from peak-5 min to post-5 min, ES = effect size, ± 99%CI = 99% confidence interval, TD = Total distance, HIRD = High-intensity running distance, HIRE = HIR effort, DrtnpHIR = Duration time of HIR, AvspdHIR = Average speed of HIR, AvlnthHIR = Average length of HIR; Sprint = Sprint distance.

### Technical Performance

**FIG. 1:**
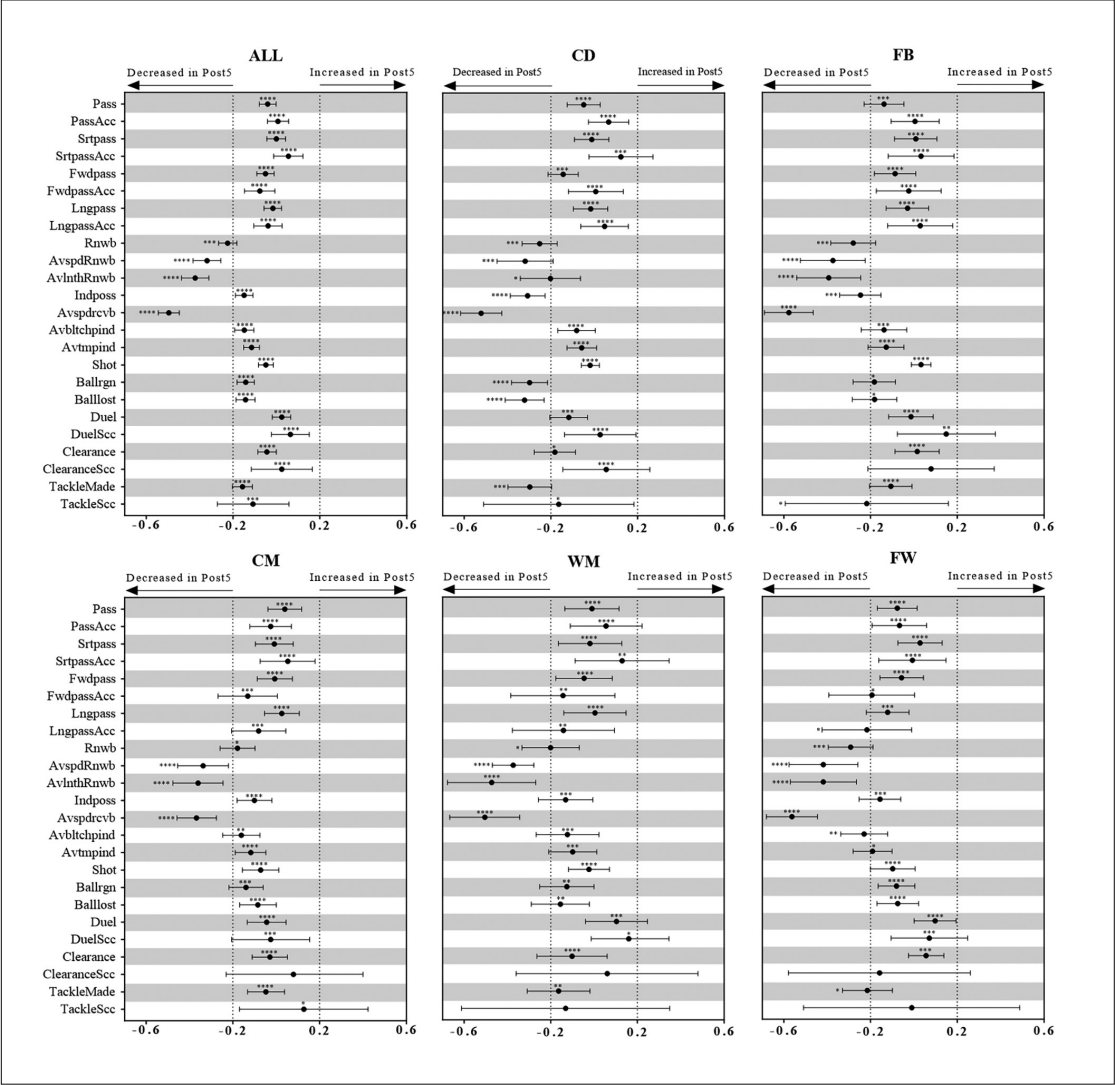
Standardized mean changes in technical performance from Peak5 to Post5 estimated from the generalized mixed linear modelling. Bars are 99% confidence intervals. Asterisks indicate the likelihood for the magnitude of the true change as follows: *possible; **likely; ***very likely; ****most likely. Asterisks located in the area between -0.2 and 0.2 denote trivial changes.

Descriptive statistics of variables related to technical performance in the Peak5 and Post5 periods estimated from the generalized mixed linear modelling are presented in Table 3, and the estimated standardized mean changes from Peak5 to Post5 are shown in Figure 1. As shown in the figure, when analyzing all the players together, substantial decrements in Rnwb, AvspdRnwb, AvlnthRnwb, and Avspdrcvb were observed, while only trivial changes were detected in all the other technical performance-related parameters. However, when analyzing players of different positions, slightly different trends have been found. Apart from Rnwb, AvspdRnwb, AvlnthRnwb, and Avspdrcvb, Indposs is also deceased in Post 5 for the CD and FB players. Furthermore, Ballrgn, Balllost, and TackleMade for the CD players decreased as well in the Post 5. While for the CM and WM players, only AvspdRnwb, AvlnthRnwb, and Avspdrcvb showed clear substantial decrements in the Post5.

**TABLE 3:** Technical performance of all players and players of each position during effective playing time of peak5 and post5 periods

Variables	ALL	CD		FB		CM		WM		FW	
	Peak5	Post5	Peak5	Post5	Peak5	Post5	Peak5	Post5	Peak5	Post5	Peak5	Post5
Pass	0.62 ± 0.67	0.60 ± 0.66	0.66 ± 0.67	0.64 ± 0.66	0.67 ± 0.62	0.59 ± 0.59	0.70 ± 0.71	0.73 ± 0.72	0.62 ± 0.61	0.61 ± 0.60	0.42 ± 0.65	0.39 ± 0.63

PassAcc	80.3 ± 32.0	80.5 ± 32.1	81.5 ± 29.5	83 ± 30	81 ± 31	81 ± 31	83 ± 31	82 ± 30	81 ± 32	83 ± 32	75 ± 39	72 ± 38

Srtpass	0.21 ± 0.44	0.21 ± 0.44	0.14 ± 0.46	0.14 ± 0.46	0.22 ± 0.43	0.23 ± 0.43	0.26 ± 0.47	0.26 ± 0.47	0.24 ± 0.43	0.24 ± 0.42	0.22 ± 0.41	0.23 ± 0.42

SrtpassAcc	78 ± 38	80 ± 38	81 ± 36	85 ± 36	83 ± 34	85 ± 35	79 ± 36	81 ± 36	75 ± 40	80 ± 41	69.1 ± 44.1	68.8 ± 44

Fwdpass	0.19 ± 0.42	0.18 ± 0.41	8.8 ± 9.7	0.82 ± 8.3	0.24 ± 0.41	0.22 ± 0.40	0.21 ± 0.41	0.20 ± 0.41	0.17 ± 0.39	0.16 ± 0.39	0.15 ± 0.46	0.13 ± 0.45

FwdpassAcc	76 ± 45	73 ± 44	79 ± 42	80 ± 42	78 ± 43	77 ± 43	76 ± 45	71 ± 43	74 ± 48	68 ± 46	68 ± 51	59 ± 48

Lngpass	0.22 ± 0.43	0.21 ± 0.43	0.29 ± 0.45	0.29 ± 0.45	0.23 ± 0.43	0.22 ± 0.42	0.26 ± 0.46	0.27 ± 0.46	0.21 ± 0.38	0.21 ± 0.38	0.11 ± 0.36	0.09 ± 0.36

LngpassAcc	74 ± 42	73 ± 42	75 ± 38	77 ± 39	69 ± 45	70 ± 46	79 ± 40	76 ± 39	79 ± 47	72 ± 45	72 ± 48	63 ± 45

Rnwb	0.61 ± 1.11	0.45 ± 1.01	1.21 ± 2.6	0.83 ± 2.38	0.58 ± 0.97	0.40 ± 0.88	0.75 ± 1.2	0.60 ± 1.2	0.72 ± 1.07	0.56 ± 0.98	0.72 ± 1.31	0.49 ± 1.18

AvspdRnwb	3.9 ± 3.1	3.1 ± 2.8	10.3 ± 6.1	8.6 ± 5.6	3.9 ± 3.1	3.0 ± 2.8	3.7 ± 2.6	3.0 ± 2.4	3.92 ± 2.64	3.08 ± 2.79	4.3 ± 3.0	3.2 ± 2.7

AvlnthRnwb	11.6 ± 6.6	9.5 ± 6.0	4.3 ± 3.1	3.8 ± 2.9	11.2 ± 6.8	8.9 ± 6.07	11.4 ± 6.2	9.5 ± 5.7	12.8 ± 6.8	10.1 ± 6.1	13.6 ± 7.1	11.1 ± 6.4

Indposs	1.12 ± 0.99	0.99 ± 0.94	3.0 ± 2.5	2.4 ± 2.2	1.2 ± 0.87	1.0 ± 0.8	1.3 ± 1.05	1.2 ± 1.01	1.1 ± 0.9	1.0 ± 0.9	1.0 ± 1.2	0.9 ± 1.1

Avspdrcvb	11.1 ± 5.0	8.9 ± 4.5	10.3 ± 4.6	8.2 ± 4.2	10.8 ± 5.0	8.4 ± 4.4	10.4 ± 4.5	8.9 ± 4.1	11.8 ± 5.2	9.5 ± 4.6	12.9 ± 5.5	10.2 ± 4.9

Avbltchpind	2.2 ± 1.1	2.0 ± 1.0	2.0 ± 0.88	1.9 ± 0.87	2.1 ± 0.94	2.0 ± 0.91	2.3 ± 1.1	2.1 ± 1.1	2.3 ± 1.1	2.2 ± 1.1	2.4 ± 1.4	2.1 ± 1.3

Avtmpind	1.6 ± 1.9	1.4 ± 1.8	1.4 ± 1.8	1.3 ± 1.7	1.5 ± 1.7	1.3 ± 1.6	1.7 ± 1.8	1.5 ± 1.7	1.7 ± 1.9	1.6 ± 1.8	1.8 ± 2.1	1.5 ± 1.9

Shot	0.012 ± 0.47	0.01 ± 0.60	0.003 ± 9.718	0.003 ± 16.5	0.001 ± 2.8	0.002 ± 1.36	0.023 ± 0.51	0.017 ± 0.67	0.01 ± 0.40	0.009 ± 0.44	0.04 ± 0.40	0.03 ± 0.47

Ballrgn	0.14 ± 0.40	0.11 ± 0.40	0.62 ± 1.02	0.42 ± 0.93	0.15 ± 0.35	0.12 ± 0.33	0.13 ± 0.39	0.10 ± 0.38	0.12 ± 0.37	0.09 ± 0.37	0.07 ± 0.48	0.06 ± 0.52

Balllost	0.21 ± 0.41	0.17 ± 0.39	0.54 ± 0.99	0.35 ± 0.92	0.23 ± 0.42	0.18 ± 0.40	0.21 ± 0.39	0.18 ± 0.38	0.20 ± 0.37	0.16 ± 0.35	0.20 ± 0.44	0.18 ± 0.43

Duel	0.11 ± 0.41	0.12 ± 0.41	0.33 ± 0.89	0.27 ± 0.88	0.085 ± 0.34	0.082 ± 0.34	0.11 ± 0.38	0.10 ± 0.38	0.09 ± 0.40	0.11 ± 0.39	0.15 ± 0.49	0.18 ± 0.50

DuelScc	50 ± 55	53 ± 56	54 ± 54	55 ± 55	51 ± 55	58 ± 58	51 ± 56	50 ± 55	42 ± 48	50 ± 54	46 ± 54	49 ± 56

Clearance	0.042 ± 0.36	0.037 ± 0.38	0.26 ± 0.93	0.2 ± 1.0	0.051 ± 0.372	0.05 ± 0.37	0.03 ± 0.41	0.02 ± 0.44	0.03 ± 0.45	0.02 ± 0.59	0.011 ± 0.65	0.014 ± 0.48

ClearanceScc	28 ± 63	29 ± 64	30 ± 60	32 ± 61	24 ± 66	27 ± 67	24 ± 58	27 ± 60	15 ± 55	16 ± 59	30 ± 70	24 ± 68

TackleMade	0.031 ± 0.42	0.018 ± 0.66	0.068 ± 1.04	0.025 ± 3.420	0.028 ± 0.44	0.019 ± 0.61	0.03 ± 0.55	0.03 ± 0.64	0.04 ± 0.34	0.03 ± 0.4	0.02 ± 0.44	0.01 ± 1.05

TackleScc	59 ± 52	54 ± 50	72 ± 49	65 ± 47	59 ± 52	50 ± 49	48 ± 54	54 ± 57	53 ± 52	47 ± 50	52 ± 54	52 ± 54

Note: all the values are estimated from the generalized mixed linear modelling. PassAcc = Pass Accuracy, Srtpass = Short pass, SrtpassAcc = Short pass Accuracy, Fwdpass = Forward pass, FwdpassAcc = Forward pass Accuracy, Lngpass = Long pass, LngpassAcc = Long pass Accuracy, Rnwb = Running with ball, AvspdRnwb = Average speed of running with ball, AvlnthRnwb = Average length of running with ball, Inposs = Individual possession, Avspdrcvb = Average speed receiving the ball, Avbltchpind = Average touches per individual possession, Avtmpind = Average time per individual possession, Ballrgn = Ball regain, DuelScc = Duel Success, ClearanceScc = Clearance Success, ClearanceScc = Clearance Success.

## DISCUSSION

This study aims to quantify the changes in physical and technical match performance in the effective playing time from the Peak5 to the Post5 in CSL soccer players. The main findings were: (I) in comparison to the Peak5 period, players experienced a notable decline in HIRD, HIRE, and Sprint during the Post5 period, and a slight decrease in TD, AvdrtnHIR, AvspdHIR, and AvlnthHIR, and players of different positions shared similar profiles; (II) analyzing all players together, substantial decreases in Rnwb, AvspdRnwb, AvlnthRnwb, and Avspdrcvb were observed, while only trivial changes were reported in all other technical performance-related parameters; (III) analyzing players of different positions, there was a clear decline in Inposs, Ballrgn, Balllost and Tacklemade for CD, and a meaningful decrement in Inposs for FB in the Post5 period.

The Peak5 in this study is identified by the rolling 5 min with the most distance covered at HIR for each player, hence it is reasonable that HIRD, HIRE, and Sprint showed a very large decrease in the Post5. Although this study only included running data within the effective playing time, the results are similar with previous studies without excluding the game interruptions [12–14, 30]. The despaired performance observed is likely caused by metabolites accumulating and enzymes inhibiting during consecutive high-intensity activity without adequate recovery [31, 32]. Schimpchen and collegues [14] found that HIRD decreased significantly in the first minute after peak intensity, ranging from 64% to 89%, and gradually decreased to 6%-31% by the fifth minute compared to the average period. This suggests that the temporary decrease in running performance will require a minimum of five minutes to recuperate [13]. As many decisive actions in football matches are associated with HIR, players may struggle to achieve optimal tactical positioning for creating offensive scoring opportunities and defensively preventing them due to temporary declines in physical performance [14]. Hence, it is necessary to employ an effective recovery strategy, which may be active resting and adaptation as shown in previous research [33].

Our results also identified a small reduction in TD during the 5 minutes following the WCS. Ju and collegues [21] found that players covered 20–55% less HIRD on ‘Covering’ and ‘Recovery run’ in the subsequent periods of WCS compared to the average period, which indicates that players tend to maintain playing formation by running at low to moderate intensity during temporary declines in running performance. The alternative view is that the interruption of play in the sequent period than in the Peak5 period, allows players to make adjustments and recover [20]. Due to the considerable variability in WCS between matches, determining the cause of a temporary decline in running performance, such as fatigue, tactical changes, pacing strategy, or interruptions during a match, is challenging without considering the specific situation [4, 34].

Furthermore, our study also identified decreases in duration, average length, and average velocity of HIR in the Post5. This means that not only did the total volume of HIR decrease after the WCS period but also the intensity of each HIR decreased accordingly. Considering the close link between HIR and tactical behaviors executed by players, a decrease in single-instance HIR distance, speed, and duration in the following periods could lead to a reduced effectiveness of specific tactical behaviors for players in certain positions, thereby changing the game’s progression. For example, a fullback might reduce their involvement in offensive plays to ensure the maintenance of their physical and tactical performance, which could result in a diminished role in the attack in their Post5 period [21].

In terms of technical performance indicators, a substantial decrease was observed in Rnwb, AvspdRnwb, AvlnthRnwb, and Avspdrcvb when combined for all players. These four technical indicators are intricately linked to running performance. Consistent with the results of the current study, Ju and collegues [21] noted a decline in the distance covered by players during ‘Run with Ball’ by 28–91% post-peak period compared to the average period. The decrease in running with the ball may be attributed to higher energy expenditure and shorter duration in comparison to running without the ball [35, 36]. When the physical condition of players temporarily declines, dribbling the ball is more likely to lose possession, hence the decrement of AvspdRnwb and AvlnthRnwb may lead players to prefer tactical cooperation with teammates over attempting individual breakthroughs [36]. In addition, Move to receive means that player moves to receive a pass from a teammate or to exploit space, which necessitates rapid alterations in direction and velocity, as well as the execution of successive acceleration and deceleration maneuvers [21]. Prior studies revealed that acceleration and deceleration activities constituted 16% of the overall player load, with a 10.4% decrease in acceleration after the peak period [37]. Move to receive typically occurs during quick transitions from defense to attack, while a decrease in average speed to receive (Avspdrcvb) may affect the team’s attacking speed and efficiency [21].

The passing-related parameters, Duel, Clearance, Shot, and TackleScc remained unaffected. These parameters may be attributed more to technical skills which are related more to the level of technique and speed and precision of decision-making rather than the physical capacities [20]. Soccer players self-regulate their match-play efforts to prevent unsustainable changes in any single physiological and psychological system [38]. Edwards and Noakes [39] suggested that soccer players adjust their effort levels based on conscious behavioral decisions informed by subconscious strategies developed through pre-match and in-match dynamics. For example, after intense play, players may choose to cover the movement of opponents instead of intercepting in defense or opt to pass the ball instead of dribbling in attacking.

It was also observed that variations in technical performance in the sequent period of WCS were position-specific, such as a decrease in Tacklemade by CD and interceptions by CD and FB. Tackles require a quick and precise strike, demonstrating a strong association with actions related to HIR, requiring players to possess exceptional anticipation in judging both ball velocity and opponent positioning [40]. The primary responsibilities of the CD are defensive, as HIRE decreases after WCS, there are fewer technical plays. Apart from overlap in FB, both CD and FB primarily perform in the backfield in terms of Indposs [21].

Several limitations warrant consideration in this study. Specifically, the analysis was limited to the initial 5 minutes post-WCS in terms of physical output and technical performance changes. Given the inherent variability of HIR, future research could expand upon this by examining additional sequence peak intensities (1 min, 3 min, 10 min, etc.) and their corresponding time frames to assess potential alterations in performance. Moreover, the study did not include analyses of acceleration and deceleration. Although these actions may not meet the criteria for HIR, they occur frequently during matches and are correlated with decreases in physical performance [30, 37, 41]. Therefore, it is essential to incorporate these actions to comprehensively assess the physical demands placed on players during matches. Furthermore, the research examined player data across various positions within different match formations, such as 1-4-4-2 and 1-4-5-1, potentially impacting the outcomes due to the influence of formation on match performance [42]. Consequently, it is imperative to evaluate the effects of match formation or playing style on technical performance during matches.

## CONCLUSIONS

We conclude that, in the initial 5 minutes post the WCS, there is a notable decline in the distance covered and efforts made in HIR, and a small decrement in the average duration, speed, and length of each high-intensity effort. These disparities in physical output have been observed in players of all positions. When analyzing all players together, there is a substantial decline in physical-related technical parameters (e.g., running with the ball, average speed receiving the ball) in the initial 5 minutes post the WCS, however, there is no meaningful change in other technical performance-related parameters. Nevertheless, there are position-specific changes in the technical performance. Specifically, there was a clear decline in the number of Inposs, Ballrgn, Balllost, and Tacklemade for CD, and a meaningful decrement in the number of Inposs for FB in the initial 5 minutes post the WCS.

## Practical applications

This study provides valuable insights for coaches to design specific technical training sessions and regulate training loads tailored to the characteristics of WCS of different player positions. In practice, beyond enhancing single-instance high-intensity running capabilities by simulating WCS in match, it is crucial to develop technical drills that align with tactical roles of players. For example, situations could be created for full backs to execute interception skills under WCS conditions, and to receive the ball at a relatively high speed in the following period. Additionally, when designing training sessions, coaches should consider players’ pacing strategies and adjust various factors, such as duration, pitch size, number of players, and rule constrictions, as needed to achieve the desired intensity simulating the WCS and post period. Specifically, the pitch size and duration of a task should allow players to reach their peak physical output like the WCS in match, meanwhile, some rule constrictions should be implemented to maintain some specific characteristics of physical performance (e.g., length, duration, speed of HIR) in the following phase.
